# Childhood Obesity and Congenital Heart Disease: A Lifelong Struggle

**DOI:** 10.3390/jcm12196249

**Published:** 2023-09-28

**Authors:** Giovanni Di Salvo, Irene Cattapan, Jennifer Fumanelli, Alice Pozza, Sara Moscatelli, Jolanda Sabatino, Martina Avesani, Elena Reffo, Domenico Sirico, Biagio Castaldi, Alessia Cerutti, Roberta Biffanti, Valeria Pergola

**Affiliations:** 1 Paediatric Cardiology and Congenital Heart Disease Complex Unit, Department of Women’s and Child’s Health, University of Padua, 35122 Padua, Italy; irene.cattapan@aopd.veneto.it (I.C.); jennifer.fumanelli@aopd.veneto.it (J.F.); alice.pozza@aopd.veneto.it (A.P.); jolanda.sabatino@unipd.it (J.S.); martina.avesani@aopd.veneto.it (M.A.); elena.reffo@aopd.veneto.it (E.R.); domenico.sirico@aopd.veneto.it (D.S.); biagio.castaldi@aopd.veneto.it (B.C.); alessia.cerutti@aopd.veneto.it (A.C.); roberta.biffanti@aopd.veneto.it (R.B.); 2 Working Group on Congenital Heart Disease and Cardiovascular Prevention in Children, Italian Society of Cardiology (SIC), 00198 Rome, Italy; s.moscatelli@rbht.nhs.uk; 3 Inherited Cardiovascular Diseases, Great Ormond Street Hospital for Children NHS Foundation Trust, London WC1N 3JH, UK; 4 Cardiology Unit, Department of Cardio-Thoraco-Vascular Sciences and Public Health, University of Padua, 35122 Padua, Italy; valeria.pergola@unipd.it

**Keywords:** childhood obesity, congenital heart disease, cardiac function

## Abstract

Congenital heart disease (CHD) affects approximately one in every one hundred infants worldwide, making it one of the most prevalent birth abnormalities globally. Despite advances in medical technology and treatment choices, CHD remains a significant health issue and necessitates specialized care throughout an individual′s life. Childhood obesity has emerged as a novel global epidemic, becoming a major public health issue, particularly in individuals with lifelong conditions such as CHD. Obesity has profound effects on cardiac hemodynamics and morphology, emphasizing the importance of addressing obesity as a significant risk factor for cardiovascular health. Obesity-induced alterations in cardiac function can have significant implications for cardiovascular health and may contribute to the increased risk of heart-related complications in obese individuals. Moreover, while diastolic dysfunction may be less apparent in obese children compared to adults, certain parameters do indicate changes in early left ventricular relaxation, suggesting that obesity can cause cardiac dysfunction even in pediatric populations. As most children with CHD now survive into adulthood, there is also concern about environmental and behavioral health risk factors in this particular patient group. Addressing obesity in individuals with CHD is essential to optimize their cardiovascular health and overall quality of life. This review aims to succinctly present the data on the impact of obesity on CHD and to enhance awareness of this perilous association among patients, families, and healthcare providers.

## 1. Introduction

Congenital heart diseases (CHD) represent a significant burden, affecting around 1.35 to 1.5 million children globally each year, equivalent to approximately one in every one hundred babies [1]. Remarkably, over 90% of children with CHD now survive into adulthood [1]. However, adulthood brings its challenges, as adults with CHD experience reduced life expectancy, with heart failure being the leading cause of premature death [2].

Over the past three decades, the prevalence of childhood obesity has seen a substantial increase worldwide, giving rise to an obesity epidemic [3,4]. The impact of obesity on cardiovascular health is significant, with overweight and obese adults facing a 1.4 and over two times higher risk of heart failure, respectively, compared to non-obese individuals [5]. Moreover, severely obese adults have been diagnosed with obesity-related cardiomyopathy, characterized by myocardial dysfunction [6]. Recent research suggests that childhood obesity also affects heart structure and function [7,8]. Alarming findings from longitudinal cohort studies indicate that obese children are more likely to become obese adults, as demonstrated in studies like the Bogalusa Heart Study [9], the Muscatine Study [10], and the Cardiovascular Risk in Young Finns Study [11].

The occurrence of malnutrition during early life has been found to have both direct and indirect associations with several illnesses that manifest in adulthood, ultimately giving rise to the idea of epigenetic memory. Experimental studies showed that already during lactation, epigenetic modifications may occur, which subsequently exert an influence on the probability of developing obesity later in life [12,13,14]. 

Obesity may already play a role on the cardiovascular system during fetal life. A study involving a population of 2,050,491 live singleton foetuses born between 1992 and 2012, demonstrated that the occurrence of different congenital heart defects, including tetralogy of Fallot, transposition of the great arteries, and atrioventricular septal defects, was significantly greater in the obese group [15]. A metanalysis of 24 studies found a direct correlation between the degree of maternal BMI and the incidence of CHDs in offspring [16]. Alterations in the availability of glucose, lipids, and proteins over the course of foetal development have the potential to impact the formation of the heart and elevate the susceptibility to cardiovascular disorders in the future. Nevertheless, the specific impacts of these associations remain uncertain and this requires further studies [17]. 

The impact of overweight and obesity is particularly critical in individuals with pre-existing heart conditions, such as children with CHD, as obesity′s adverse effects on the cardiovascular system may exacerbate existing issues [18,19]. A recent review highlighted obesity and overweight prevalence in children with CHD reaching up to 22% and 36%, respectively [20]. Contributing factors include physical activity restrictions and interventions promoting weight gain in infancy [3]. Regrettably, unhealthy behaviors are often fostered within families, leading to limited physical activity and unhealthy lifestyles, including poor dietary habits [3]. Demographic factors also play a role, with males, minorities, publicly insured individuals, and those with Down syndrome and asthma being more susceptible to obesity′s impact on CHD patients [21]. Interestingly, complex CHD patients initially exhibit lower obesity rates as young children, which progressively increase over time to align with the general population [21]. Importantly, these patients often face chronic illness and suffer from residuals and sequelae of the underlying cardiac disorder, previous surgeries, or interventional procedures [22].

Early interventions and family education on healthy lifestyles are key components in addressing obesity in this vulnerable population and improving long-term outcomes. By focusing on prevention and early intervention strategies, we can enhance the lives of children with CHD and foster better cardiovascular health throughout their lifespan.

This review aims to succinctly present the data on the impact of obesity on CHD and to enhance awareness of this perilous association among patients, families, and healthcare providers. 

## 2. Impact on Cardiac Hemodynamics

Overall, obesity’s impact on cardiac function is significant, affecting both left and right ventricles, and can have significant implications, especially when combined with pre-existing congenital heart diseases. Obesity has profound effects on cardiac hemodynamics and morphology. Obesity leads to an increase in both total and central blood volume, resulting in elevated cardiac output (CO). This elevation in CO is primarily due to an increase in left ventricular (LV) stroke volume and a reduction in peripheral vascular resistances [19,23]. Moreover, severely obese individuals commonly exhibit heightened oxygen consumption [24]. This can be accompanied by elevated LV end-diastolic and pulmonary capillary wedge pressures. Additionally, class II–III obese patients may experience higher right heart pressures and pulmonary vascular resistance compared to what would be predicted for individuals with normal weight.

Studies involving autopsies, ultrasounds, and cardiac magnetic resonance imaging (cMRI) have provided insights into the effects of obesity on cardiac morphology [25,26]. One of the most frequently observed alterations is LV hypertrophy, often eccentric, particularly in the absence of concurrent hypertension. Furthermore, epicardial fat tends to increase in individuals with obesity [27].

In normotensive obese subjects, left atrial enlargement is a common finding and represents a major risk factor for left atrial enlargement and atrial arrhythmias during aging [22,28].

A study by Chahal et al. called the MESA—Right Ventricular Study used cMRI to explore the relationship between overweight/obesity and right ventricular structure. The study involved 4127 patients, and it revealed that the right ventricular mass was 15% greater in overweight/obese patients compared to lean patients (*p* < 0.001 for the trend). Additionally, right ventricular volumes were 26% larger in overweight/obese subjects compared to lean patients (*p* < 0.001 for the trend) [29].

## 3. Impact of Obesity on Cardiac Function

Obese children often exhibit a lower LV ejection fraction compared to their lean counterparts [7,8]. Moreover, irrespective of blood pressure levels, obese children tend to have thicker LV mass [30].

Doppler echocardiography studies have revealed higher LV stroke volumes and cardiac output at rest in obese children, likely due to their increased metabolic demands. The larger LV with a normal ejection fraction compensates for the chronic metabolic demands induced by obesity [31]. However, in the presence of concomitant shunt lesions associated with volume overload, such as ventricular septal defects or patent ductus arteriosus, these hemodynamic consequences of obesity may exacerbate the existing issues [32,33]. 

Assessing myocardial deformation using imaging techniques has proven valuable for detecting early changes in myocardial mechanics. Several studies have consistently shown that obese children have reduced global and regional longitudinal systolic strain and strain rate values compared to normal weight controls [25,34,35].

While data on right ventricular (RV) myocardial deformation in obese children are limited and conflicting, some studies have reported reduced longitudinal systolic strain values in the RV free wall using cardiac tissue Doppler imaging (CTDI) techniques [36]. However, another study by Barbosa et al. found higher values [37]. Interestingly, tricuspid valve annular plane systolic excursion (TAPSE) appears to be normal in obese children [38,39,40].

Recent cMRI studies on obese children have confirmed impairment of right ventricular systolic function [41]. Moreover, Xu et al. [42] studied 45 children with severe obesity and found that children and adolescents may present early and subclinical cardiac impairment detected by multichamber cMRI. Children with obesity showed reduced LA reservoir and contraction phase strain, dilated RV with altered RV strain, and higher LVMI and LV average wall thickness with lower LVEF. RV systolic function was also impaired, with an altered RV free wall circumferential strain and radial motion fraction.

Obesity can significantly impact LA function, as well. LA enlargement is a common finding in obese individuals, attributed to accommodating the increased blood volume and handling higher metabolic demands [6]. However, LA enlargement can lead to impaired left atrial function, particularly reduced compliance and reservoir function [43].

Additionally, obesity is associated with increased left atrial stiffness, compromising LV filling during diastole and potentially leading to diastolic dysfunction [43,44,45,46]. These changes in LA function are not limited to adults but have also been observed in obese children, even in the absence of other cardiovascular risk factors [45].

## 4. Conventional Diastolic Function Parameters

In children, the impact of obesity on filling pressures and diastolic function is not as pronounced as in adults. While diastolic dysfunction is often evident in obese adult patients, studies involving obese children have shown less consistent changes in early filling characteristics, atrial contraction inflow velocities, or E/A ratio [25,45]. However, certain diastolic parameters do exhibit alterations in obese children.

Notably, studies have revealed that obese children may experience an increased isovolumic relaxation time, indicating impaired early left ventricular relaxation [46]. Additionally, the mitral valve inflow deceleration time is often prolonged in obese children, potentially leading to delayed left ventricular relaxation [47]. However, findings related to pulmonary venous flow patterns in children with obesity have been inconsistent, likely due to technical challenges in obtaining accurate data [47,48].

Regarding the inflow patterns of the tricuspid valve, limited research has been conducted on this specific aspect in obese children. However, most available studies have not found significant differences between obese children and controls in this regard [47,48,49,50].

While the impact of obesity on diastolic function in children may be less apparent than in adults, these subtle changes in diastolic parameters can still have implications for cardiac health which requires monitoring, as early detection and management of diastolic function may optimize patients’ cardiovascular well-being.

## 5. Obesity and Arrhythmias

Obesity has been linked to an increased risk of atrial fibrillation (AF) in patients with congenital heart disease (CHD), although the precise mechanisms underlying this association remain unclear. In an animal study involving sheep fed a high-calorie diet, researchers observed that increased body weight led to enlargement of the left atrium, along with fibrosis, inflammation, and lipidosis in the atria. Despite these changes, there were no significant alterations in certain electrical properties that would directly induce AF. However, specific molecular markers in the atria were significantly elevated with increasing body fat, suggesting that obesity may contribute as an additional risk factor for arrhythmias in CHD patients [51].

In individuals with CHD, obesity can induce both electrical and structural changes in the heart, creating a pro-arrhythmic environment [51,52]. Research has demonstrated a higher prevalence of AF and ventricular arrhythmias in obese CHD patients compared to those with normal weight [52,53]. Obesity-related alterations in cardiac structure and function, such as LA enlargement and LV hypertrophy, play a role in promoting atrial and ventricular arrhythmias [43,51,52,53,54]. Additionally, obesity is associated with impaired autonomic regulation, increased sympathetic activity, and altered parasympathetic tone, further predisposing CHD patients to arrhythmias [55]. Furthermore, obesity-related inflammation and oxidative stress may contribute to the development of arrhythmias in this patient population [56].

Particular attention should be given to managing AF in obese patients with CHD, as it can significantly impact their health outcomes [54]. Effective management strategies for arrhythmias in obese CHD patients should encompass lifestyle modifications to promote weight loss and improve overall cardiovascular health [55]. Additionally, close monitoring and aggressive management of other cardiovascular risk factors, such as hypertension and dyslipidemia, are crucial in reducing the incidence and progression of arrhythmias in this vulnerable group [56]. 

## 6. Obesity, Inflammation, and Atherogenesis in Congenital Heart Disease

Obesity, inflammation, and atherogenesis form a complex interplay with profound implications for individuals with CHD [57]. Obesity, characterized by excessive accumulation of body fat, triggers chronic low-grade inflammation throughout the body [58]. In the context of CHD, obesity can further amplify the underlying inflammatory response, fostering atherogenesis, the formation of fatty plaques in the arteries [59].

Children and adults with CHD face an increased risk of developing obesity due to factors like limited physical activity, sedentary lifestyle, and potential complications of the heart condition itself [59]. In CHD patients, obesity induces an inflammatory cascade as adipose tissue releases pro-inflammatory cytokines and chemokines [60,61]. This persistent state of inflammation can accelerate atherosclerosis, including inflammation of peri-coronary fat [62].

Atherosclerosis poses significant health hazards for CHD patients, leading to coronary artery disease, heart attacks, strokes, and other cardiovascular complications [63]. Moreover, obesity can detrimentally impact endothelial function, promoting endothelial dysfunction, and further contributing to atherogenesis [59].

Research has demonstrated that obese children and adults with CHD exhibit elevated levels of inflammatory markers, such as C-reactive protein (CRP) and interleukin-6 (IL-6), which are associated with heightened cardiovascular risk [64]. These inflammatory markers can be particularly elevated in the presence of additional risk factors like hypertension, dyslipidemia, and insulin resistance, commonly seen in obese CHD patients [65].

To counteract the adverse effects of obesity, inflammation, and atherogenesis in CHD patients, a comprehensive approach is indispensable. This entails lifestyle modifications, encouraging healthy eating habits, regular physical activity, and weight management, alongside vigilant monitoring and management of cardiovascular risk factors [66]. Furthermore, early detection of obesity and inflammation in CHD patients enables targeted interventions to reduce the risk of atherogenesis and its associated complications [66]. Table 1 outlines various aspects of management, along with the specific components and the roles of different healthcare professionals involved in each aspect. The approach emphasizes personalized care tailored to each patient′s unique needs, with a focus on promoting cardiovascular health, weight management, psychosocial support, and a smooth transition to adult congenital specialized care.

## 7. Obesity and Aortic Coartation

Long-term follow-up data in individuals with repaired aortic coarctation (CoA) reveal a persistent reduction in life expectancy, primarily attributed to late arterial hypertension and atherosclerosis [67]. A study involving 103 adult patients with aortic coarctation demonstrated that despite successful treatment, these individuals displayed significantly lower exercise tolerance compared to their healthy counterparts (*p* < 0.0001). Although their weight status was similar to the overall Belgian population, there was a tendency towards a higher body mass index. Additionally, patients with CoA reported lower levels of habitual physical activity and experienced reductions in perceived vitality, general health, and mental well-being [68].

In a recent cross-sectional study of 160 patients who underwent CoA repair between 1974 and 2009, it was evident that patients with CoA had significantly higher BMI z-scores compared to age-sex matched normal data after the age of 5 years (*p* < 0.001). Furthermore, the prevalence of obesity in these patients increased significantly over time. Compared to other individuals with congenital heart defects, adults who had their CoA repaired were found to have a higher likelihood of developing obesity [69].

Advanced echocardiographic techniques shed further light on the impact of obesity in young CoA patients even after successful treatment. Obesity in this population was linked to elevated diastolic blood pressure, LV hypertrophy, diastolic abnormalities, reduced LV twist, and myocardial deformation abnormalities. These findings are particularly concerning as life expectancy in CoA patients is primarily limited by atherosclerosis [19].

## 8. Obesity and Arterial Switch Operation

Obesity has been found to have significant effects, especially in patients who are already at risk for early atherosclerotic disease, such as those who have undergone the arterial switch operation (ASO) [70]. ASO, a surgical repair procedure, can potentially impact ventricular function [70,71].

Pasquali et al. conducted a study [72] to assess comorbidities and early cardiovascular disease markers in obese ASO patients. The results revealed that obese ASO patients exhibited higher systolic blood pressure, nighttime diastolic blood pressure, left ventricular mass index, and lower brachial artery reactivity when compared to normal weight ASO patients and controls. Additionally, obese ASO patients had increased carotid artery intima-media thickness, higher triglyceride levels, and lower high-density lipoprotein levels. These findings suggest that obesity may introduce additional risks for future cardiovascular events in this unique population, which underwent coronary artery reimplantation in infancy. Moreover, several studies have indicated that children who have undergone coronary artery reimplantation surgery are particularly susceptible to atherosclerotic-related lesions [70,73].

## 9. Obesity and Tetralogy of Fallot

Obesity poses significant challenges for individuals with Tetralogy of Fallot (TOF), impacting their cardiovascular health and overall well-being. Research has shown that obesity can exacerbate cardiovascular risk factors in TOF patients, leading to increased systemic hypertension and reduced exercise tolerance [74,75,76]. The additional strain on the heart from excess weight is particularly concerning for individuals with pre-existing cardiac abnormalities like TOF.

Moreover, obesity has been associated with a higher risk of postoperative complications after TOF repair surgery. Obese TOF patients may experience longer hospital stays, higher rates of postoperative complications, and an increased likelihood of requiring mechanical ventilation [74,75,76].

In the long term, obesity may also affect outcomes in TOF patients, with a potential increased risk of developing pulmonary hypertension, which can worsen the prognosis for this population [77,78].

Following successful repair, obesity in TOF patients has been linked to several adverse effects on cardiovascular health and functional outcomes. Obese TOF patients often have a lower New York Heart Association (NYHA) class, indicating poorer functional status compared to normal-weight patients [78]. Additionally, cMRI has revealed that obesity in TOF patients is associated with increased biventricular volumes, reduced RV ejection fraction, and LV ejection fraction [79].

Exercise performance is also affected in obese TOF patients, which can have implications for their overall quality of life and functional capacity [80]. These findings highlight the importance of addressing obesity as part of the management of TOF patients after successful repair.

A recent study by Ghonim et al. [81] underscored the significance of considering both lesion-specific characteristics and individual patient factors in predicting survival outcomes in repaired TOF patients. This personalized approach can guide clinicians in understanding the prognosis of individual patients, enabling more targeted management and follow-up care.

Given the adverse cardiovascular effects of obesity in TOF patients, there is a growing interest in implementing cardiac rehabilitation programs with a specific focus on weight management. Cardiac rehabilitation can provide a structured approach to improving cardiovascular health, optimizing exercise capacity, and promoting weight loss in this patient population [82].

## 10. The Impact of Obesity in Fontan Patients

Children with single ventricle physiology often face growth restrictions in terms of weight and height, potentially attributed to hemodynamic limitations, such as chronic reduction in cardiac output and oxygen delivery [83]. However, as Fontan patients reach adulthood, the prevalence of overweight and obesity in this population approaches that of the general population, with 39% classified as overweight or obese and 17% falling into the obese category [84]. Notably, individuals with a BMI ≥ 85th percentile during childhood have a three-fold higher likelihood of being overweight or obese as adults (CI 2.1–4.5, *p* < 0.01) [84].

Moreover, studies have highlighted that increased adiposity in Fontan patients is associated with a heightened risk of cardiovascular events [84,85], underscoring the significance of addressing weight management in this vulnerable population.

Fascinatingly, a subgroup of Fontan patients has been identified as “Super-Fontans”, exhibiting remarkable resilience under stress despite lacking the subpulmonary ventricle in the Fontan circulation [86]. These “Super-Fontans” tend to undergo the Fontan procedure earlier, engage in more frequent exercise during childhood, and maintain a healthy weight. The discovery of the “Super-Fontan” phenotype underscores the importance of promoting physical activity, sports participation, exercise training, and weight control from an early age in individuals with Fontan circulation [86]. 

## 11. The Impact of Obesity on Pulmonary Arterial Hypertension and Untreated Shunt Lesions

West et al. examined the correlation between BMPR2 and pulmonary arterial hypertension (PAH), revealing that the activation of BMPR2 mutation was linked to the occurrence of insulin resistance at an early stage [87]. There is also the potential for the existence of additional unidentified genes that may confer a predisposition in patients towards a rapid onset of insulin resistance, followed by the development of pulmonary arterial hypertension [88]. 

Nevertheless, it is reasonable to consider insulin resistance as a consequence of chronic hypoxic injury to the pancreatic beta cells [88]. Indeed, it has been reported that hypoxia secondary to atherosclerosis has the potential to adversely affect beta cells [89]. Similarly, the occurrence of hypoxia resulting from severe PAH or the existence of a right-to-left shunt can also contribute to the impairment of beta cells and subsequent development of prediabetic or diabetic states, potentially exacerbating PAH.

There are many evidences suggesting that the prediabetic state would exacerbate the impairment of the pulmonary vascular system and have a role in the advancement of PAH [88]. 

Indeed, in experimental models, it has been demonstrated that high glucose levels potentiate migration and proliferation of smooth muscle cells [88,90,91].

The aforementioned elements are key components linked to vascular remodeling, leading to an elevation in pulmonary vascular resistance. Therefore, in the case of individuals experiencing prolonged left-to-right shunting, it is plausible that insulin resistance or prediabetes may act as a secondary factor that expedites the initiation or advancement of pulmonary vascular disease and PAH [88,92]. 

## 12. Strategies for Prevention of Obesity in CHD

The Commission on Ending Childhood Obesity was created by WHO in 2014. The Commission issued six specific suggestions in 2016 to address the environmental factors that are obesogenic for children from conception through adolescence [93]. 

According to the WHO Global Nutrition Policy Review 2016–2017, which relies on self-reported data from national governments and is subsequently confirmed by the WHO, nearly 80% of the 167 countries surveyed have implemented nutrition-related measures aimed at addressing the issue of childhood obesity. Nevertheless, a mere 16 percent of nations had established regulations pertaining to the marketing of complementary foods, while 40 percent had implemented laws aimed at controlling the marketing of food and non-alcoholic beverages, specifically targeted towards children [94].

As of the conclusion of 2018, over 40 nations had commenced the implementation of a kind of taxation on beverages containing high levels of added sugars, which is recognized as one of the World Health Organization′s recommended cost-effective interventions [95,96]. 

Given the alarming rates of excess weight among CHD patients, assessments of diet and physical activity as suggested by WHO should become an integral part of routine care. Since children with CHD solely rely on parental guidance, parents need to be adequately educated and trained in taking care of their cardiopathic child in order to prevent common weight misperceptions. In addition to nutritional guidance, psychological counseling might be necessary for both, parents and patients, to reduce psychological distress and facilitate coping with the disease [97]. 

The prevention and management of obesity in patients with CHD require a multifaceted approach that addresses both lifestyle choices and personalized exercise activities. Education and awareness play a crucial role in empowering patients and their families to make informed decisions and adopt healthy habits [98]. This collaborative effort among healthcare professionals, including cardiologists, dietitians, sports doctors, and psychologists, ensures that comprehensive treatment plans are tailored to each individual′s unique needs [98]. Table 2 describes a strategy for planning and monitoring the well-being of patients with CHD. Tailored exercise activities, carefully designed to accommodate medical history, physical limitations, and health goals, are pivotal in effectively managing weight and promoting overall well-being [99]. By closely collaborating with healthcare professionals, young patients with CHD can engage in safe and effective exercise routines that enhance their cardiovascular health [99]. Incorporating enjoyable activities such as swimming or cycling fosters a positive attitude towards exercise, encouraging long-term adherence to healthy habits.

## 13. The Importance of a Dedicated Adult Congenital Cardiology Team 

The establishment of a dedicated adult congenital cardiology team is of paramount importance in providing comprehensive and specialized care for patients with CHD as they transition from childhood to adulthood. This specialized team consists of healthcare professionals with expertise in both pediatric and adult cardiology, ensuring that the unique needs of adult CHD patients are met throughout their lifespan [100,101,102].

Adult CHD patients require specialized care that may differ significantly from the management of acquired heart conditions in adults. The dedicated adult congenital cardiology team possesses in-depth knowledge and experience in treating complex congenital heart defects, understanding the nuances of congenital anatomy, and managing the long-term sequelae of CHD.

The transition from pediatric to adult care can be challenging for CHD patients. A dedicated adult congenital cardiology team facilitates a smooth transition process, ensuring continuity of care and minimizing disruptions in medical management. This seamless transition is crucial for optimizing outcomes and preventing gaps in healthcare [100,101,102].

In contrast to many adult cardiac conditions, CHD often requires lifelong follow-up and monitoring. The adult congenital cardiology team is equipped to provide ongoing surveillance, detecting and managing complications or changes in heart function that may arise over time. Each adult CHD patient is unique, with their specific medical history, anatomy, and treatment journey. The dedicated team develops personalized treatment plans, considering the individual′s health status, lifestyle, and future goals to optimize their cardiac health and quality of life. The complexity of congenital heart disease demands a multidisciplinary approach to care. The adult congenital cardiology team collaborates with various specialists, including cardiothoracic surgeons, electrophysiologists, interventional cardiologists, and cardiac imaging experts, to provide comprehensive and integrated care for each patient.

Adult CHD patients may face psychosocial challenges related to their unique medical history. The dedicated team includes psychologists or social workers who can address emotional and psychological concerns, offering support and resources to enhance the patient′s overall well-being [102].

A dedicated adult congenital cardiology team is often involved in research and clinical trials focused on advancing the understanding and treatment of CHD. This commitment to research ensures that patients have access to the latest advancements and therapies.

The team places a strong emphasis on patient education, empowering adult CHD patients to actively participate in managing their condition. By providing knowledge and guidance, patients can make informed decisions about their health and lifestyle choices. By combining expertise, personalized treatment plans, and a multidisciplinary approach, this team ensures that adult CHD patients receive the highest level of care throughout their lives, optimizing their cardiovascular health and overall well-being.

## 14. Conclusions

In conclusion, obesity presents significant challenges for individuals with CHD. Studies have shown that obesity can exacerbate cardiovascular risk factors, lead to impaired exercise tolerance, and impose additional strain on the heart in CHD patients. Moreover, obesity may increase the risk of postoperative complications after CHD repair surgery and impact long-term outcomes, potentially leading to the development of pulmonary hypertension and affecting functional status.

The relationship between obesity and CHD is complex, with multiple factors intertwining to influence cardiovascular health and overall well-being. The impact of obesity on cardiac function, hemodynamics, and morphology in children with CHD highlights the importance of addressing weight management early in life to prevent adverse consequences in adulthood.

To effectively prevent and manage obesity in CHD patients, a multidisciplinary approach is essential. This includes education and awareness about healthy lifestyle choices, collaboration among healthcare professionals to develop tailored treatment plans, and the incorporation of enjoyable and safe exercise activities. By prioritizing these strategies, we can empower individuals with CHD to lead healthier lives, optimize their cardiovascular health, and improve their overall quality of life.

In embracing this holistic and patient-centered approach, we embark on a journey towards empowering individuals with CHD to lead fulfilling lives while effectively managing their condition, promoting better health for generations to come.

## Figures and Tables

**Table 1:** Schematic approach to multidisciplinary congenital heart disease (CHD) management.

Aspect of Management	Component	Role of Healthcare Professionals
**Cardiology Assessment**	Clinical evaluation	Cardiologist
	Echocardiography	Cardiologist, Echocardiographer
	Cardiac MRI/CT	Cardiologist, Cardiac MRI/CT Specialist
	Cardiac Catheterization	Cardiologist, Interventional Cardiologist
**Nutrition and Diet**	Dietary assessment	Dietitian
	Individualized meal planning	Dietitian
	Nutritional counseling	Dietitian
**Physical Activity**	Exercise prescription	Cardiologist, Exercise Physiologist
	Tailored exercise programs	Exercise Physiologist
	Monitoring of physical activity	Exercise Physiologist
**Weight Management**	Weight assessment	Pediatrician, Dietitian
	Weight monitoring	Pediatrician, Dietitian
	Weight management counseling	Pediatrician, Dietitian
**Psychosocial Support**	Emotional well-being support	Psychologist, Counselor
	Coping strategies	Psychologist, Counselor
	Family support and counseling	Psychologist, Counselor
**Medication Management**	Medication prescription	Cardiologist, Pediatrician
	Medication monitoring	Cardiologist, Pediatrician
	Adverse effects monitoring	Cardiologist, Pediatrician
**Monitoring of CHD Status**	Regular check-ups	Cardiologist, Pediatrician
	Periodic echocardiography	Cardiologist, Echocardiographer
	Cardiac MRI/CT follow-up	Cardiologist, Cardiac MRI/CT Specialist
**Management of Complications**	Heart failure management	Cardiologist, Pediatrician
	Arrhythmia management	Cardiologist, Electrophysiologist
	Pulmonary hypertension	Cardiologist, Pulmonologist
**Transition to Adult Care**	Transition planning	Cardiologist, Pediatrician, Transition Coordinator
	Coordination with adult care	Adult Cardiology Specialized Centre
	Health education for adults	Adult Cardiologist Specialized Centre

**Table 2:** Strategies for prevention and management of obesity in CHD patients.

Multidisciplinary Approach	A Multidisciplinary Approach Including Cardiologists, Dietitians, Exercise Physiologists, and Mental Health Specialists Allows for a Comprehensive Assessment and Personalized Interventions Tailored to the Individual Patient′s Needs.
Nutritional Counseling	Dietitians can work closely with patients and their families to create meal plans that support cardiovascular health and weight management while taking into account any dietary restrictions or considerations related to the CHD physiology.
Psychological Support	Mental health support and counseling can play a crucial role in addressing emotional eating, stress management, and fostering a positive body image.
Regular Monitoring and Follow-Up	Consistent follow-up with healthcare providers allows for early identification of potential weight-related issues and timely interventions.
Physical Activity	Exercise programs should be tailored to the individual′s capabilities and medical considerations, with an emphasis on safe and enjoyable activities.
Family Support and Education	Family involvement and support are essential for promoting healthy behaviors and lifestyle changes in Fontan patients. Educating families about the importance of nutrition and exercise and involving them in the treatment process can enhance the effectiveness of obesity management strategies.
Addressing Sedentary Behaviors	Reducing sedentary behaviors, such as excessive screen time and prolonged sitting, is crucial for preventing weight gain in CHD patients. Encouraging active play and limiting screen time can contribute to overall health improvement.
Peer Support and Group Activities	Providing opportunities for CHD patients to engage in group activities and interact with peers with similar conditions can create a supportive environment and motivate them to maintain a healthy lifestyle.
